# Vitamin B12 Deficiency and the Nervous System: Beyond Metabolic Decompensation—Comparing Biological Models and Gaining New Insights into Molecular and Cellular Mechanisms

**DOI:** 10.3390/ijms25010590

**Published:** 2024-01-02

**Authors:** Aimee Rachel Mathew, Giacomo Di Matteo, Piergiorgio La Rosa, Saviana Antonella Barbati, Luisa Mannina, Sandra Moreno, Ada Maria Tata, Virve Cavallucci, Marco Fidaleo

**Affiliations:** 1 Department of Biology and Biotechnologies “Charles Darwin”, Sapienza University of Rome, 00185 Rome, Italy; aimeerachel.mathew@uniroma1.it (A.R.M.); adamaria.tata@uniroma1.it (A.M.T.); 2 Department of Chemistry and Technology of Drugs, Sapienza University of Rome, 00185 Rome, Italy; giacomo.dimatteo@uniroma1.it (G.D.M.); luisa.mannina@uniroma1.it (L.M.); 3 Division of Neuroscience, Department of Psychology, Sapienza University of Rome, 00185 Rome, Italy; piergiorgio.larosa@uniroma1.it; 4 European Center for Brain Research, IRCCS Fondazione Santa Lucia, 00179 Rome, Italy; 5 Departmental Faculty of Medicine and Surgery, UniCamillus-Saint Camillus International University of Health Sciences, 00131 Rome, Italy; saviana.barbati@unicamillus.org; 6 Department of Science, University Roma Tre, 00146 Rome, Italy; sandra.moreno@uniroma3.it; 7 Laboratory of Neurodevelopment, Neurogenetics and Neuromolecular Biology, IRCCS Fondazione Santa Lucia, 00179 Rome, Italy; 8 Research Centre of Neurobiology “Daniel Bovet”, Sapienza University of Rome, 00185 Rome, Italy; 9 Dipartimento di Medicina e Chirurgia Traslazionale, Università Cattolica del Sacro Cuore, 00168 Rome, Italy; virve.cavallucci@unicatt.it; 10 Fondazione Policlinico Universitario “A. Gemelli” IRCCS, 00168 Rome, Italy; 11 Research Center for Nanotechnology Applied to Engineering (CNIS), Sapienza University of Rome, 00185 Rome, Italy

**Keywords:** vitamin B12, nervous system homeostasis, neurodegeneration, metabolic decompensation, energy balance, nutrients, cellular metabolism, antioxidants, oxidative stress

## Abstract

Vitamin B12 (VitB12) is a micronutrient and acts as a cofactor for fundamental biochemical reactions: the synthesis of succinyl-CoA from methylmalonyl-CoA and biotin, and the synthesis of methionine from folic acid and homocysteine. VitB12 deficiency can determine a wide range of diseases, including nervous system impairments. Although clinical evidence shows a direct role of VitB12 in neuronal homeostasis, the molecular mechanisms are yet to be characterized in depth. Earlier investigations focused on exploring the biochemical shifts resulting from a deficiency in the function of VitB12 as a coenzyme, while more recent studies propose a broader mechanism, encompassing changes at the molecular/cellular levels. Here, we explore existing study models employed to investigate the role of VitB12 in the nervous system, including the challenges inherent in replicating deficiency/supplementation in experimental settings. Moreover, we discuss the potential biochemical alterations and ensuing mechanisms that might be modified at the molecular/cellular level (such as epigenetic modifications or changes in lysosomal activity). We also address the role of VitB12 deficiency in initiating processes that contribute to nervous system deterioration, including ROS accumulation, inflammation, and demyelination. Consequently, a complex biological landscape emerges, requiring further investigative efforts to grasp the intricacies involved and identify potential therapeutic targets.

## 1. Introduction

Vitamin B12 (VitB12), also known as cobalamin (Cbl), is a water-soluble vitamin mainly found in animal foods, meat, milk, eggs, fish, and shellfish [1]. In the 1960s, the pivotal role of this micronutrient was recognized through studies on pernicious anemia, a condition directly linked to VitB12 deficiency [2]. Over the years, mild to severe alterations associated with VitB12 dysregulation have been described, even independent of vitamin intake or the amount of liver storage but mostly related to various inborn errors. Indeed, the extent of VitB12 deficiency-related issues is not only linked to low intake but also to the ability of the organism to uptake and utilize VitB12. Therefore, instead of determining the VitB12 levels, clinical practices focus on evaluating the levels of intermediate metabolites that can accumulate based on the biochemical reactions catalyzed by VitB12 (i.e., methylmalonic acid, MMA, and homocysteine, HCy) [3,4].

The range of diseases linked to VitB12 deficiency is broad, encompassing megaloblastic anemia, hypercellular and dysplastic bone marrow, growth and developmental challenges in children, infertility, thrombosis due to hyperhomocysteinemia (HHCy), and syndromes with fatal hematological and neurological symptoms [5,6,7,8]. VitB12 deficiency can give rise to a wide range of alterations, including both neurological and neuropsychological aspects, which can manifest as cognitive impairments, depression, mania, irritability, paranoia, delusions, and emotional instability [8]. Moreover, in recent years, VitB12 deficiency has been associated with neurodegenerative diseases (refer below for more information). Furthermore, disruptions in the autonomic nervous system and spinal cord accompany VitB12 deficiency, determining issues such as postural hypotension, incontinence, and impotence, while affecting the peripheral nervous system with symptoms like cutaneous sensory loss, reduced reflexes, symmetric weakness, and paraesthesia (Stabler et al., 2013; Kayali et al., 2019; Scalabrino et al., 2006 [8,9,10]).

The strategy of deficiency management aims to normalize the metabolic decompensation and primarily relies on the supplementation of VitB12 and on drugs targeting the reduction of toxic metabolite build-up. While these treatments are life-saving interventions, they often fall short of effectively addressing the underlying neurological abnormalities and only partially ameliorate symptoms caused by VitB12 deficiency. Recovery from neurological impairment poses significant challenges, primarily because the neurological dysfunction associated with VitB12 deficiency often manifests years after the onset of malabsorption or the absence of supplementation, i.e., when neurological recovery is no longer possible [11].

Inborn errors related to VitB12 uptake (e.g., Intrinsic Factor deficiency, Imerslund–Gräsbeck syndrome, transcobalamin deficiency) or intracellular VitB12 metabolism (such as methylmalonic acidemia with homocystinuria) have the potential to recapitulate and mimic the effects of VitB12 deficiency, and can provide valuable insights into the involvement of the nervous system. In these disorders, neurological impairments can manifest soon after birth. Although the diagnosis can be achieved within a few days after birth (especially in areas where metabolic postnatal screening is available, typically within 1 to 5 days), neurological impairments are not completely reversible despite prompt treatment [12]. It is essential to emphasize that VitB12 is indispensable for fetal development, although pregnancy can still progress even in the presence of VitB12 deficiency or severe inborn errors affecting its metabolism. Several factors may contribute to this phenomenon, including the exchange of reaction intermediates or end-products between the mother and fetus via the placenta and the inheritance of maternal components in the zygote (as discussed later). Furthermore, in a case report involving methylmalonic aciduria with homocystinuria as the inborn error, a combination of pre- and postnatal treatment with hydroxocobalamin (OH-Cbl) and folic acid (FA), along with postnatal treatment using OH-Cbl, folic acid, and betaine, avoided the onset of metabolic decompensation and enabled the patient to achieve a normal intelligence quotient (IQ), even though mild ophthalmic issues were still present [13]. Similarly, the offspring of mothers strictly adhering to a vegan or vegetarian diet, who breastfed their babies, exhibited neurological symptoms associated with VitB12 deficiency only during their second trimester coinciding with the onset of metabolic decompensation [14]. Furthermore, some individuals carry mutations associated with defects in VitB12 metabolism but only experience complications later in life [15]. On one hand, these observations suggest a correlation between metabolic decompensation due to a defect in VitB12 metabolism and disease symptoms. On the other hand, it is important to note that some disease-related manifestations, such as eye issues, may not be directly associated with metabolic imbalance. These observations raise questions about the possible existence of endogenous mechanisms that can counteract or buffer defects linked to VitB12, sustaining metabolic balance or postponing the onset of symptoms, or the possibility of non-biochemical but rather cellular and molecular mechanisms that could contribute to this phenomenon.

While previous research has primarily explored the biochemical alterations stemming from the defective pathways directly linked to VitB12 as a coenzyme, the investigation of secondary alterations involving molecular changes and cellular alterations is a more recent focus. Furthermore, despite the apparent link between neurological impairments and VitB12 deficiency, the possible neuropathological mechanisms have not yet been fully clarified. Therefore, in this review, we delve into the cellular processes and molecular mechanisms linked to VitB12 deficiency or VitB12 deficiency mimicking conditions (e.g., inborn errors), with a specific focus on the nervous systems. In addition, we report differences in experimental models used to investigate the role of VitB12 and highlight possible bias. Our objective is to emphasize potential mechanisms worth further investigation and to identify potential targets already described in the literature for future medical interventions, aiming for the improvement of neurological impairments due to VitB12 deficiency.

## 2. Vitamin B12 Micronutrient: As Essential as Complex

VitB12 comprises a group of four molecules distinguished by a solitary cobalt atom binding to a corrin-like core, along with 5,6-dimethylbenzimidazole (which, in turn, binds to ribose 5-phosphate) and a variable residue. This latter component encompasses one of the following groups: cyanide, hydroxyl, methyl, or 5′-deoxyadenosyl, which corresponds to cyanocobalamin (CN-Cbl), hydroxocobalamin (OH-Cbl), methylcobalamin (Me-Cbl), and 5′-deoxyadenosylcobalamin (also known as adenosylcobalamin, Ado-Cbl), respectively. OH-Cbl is the naturally occurring form of VitB12; conversely, CN-Cbl is a by-product of the extraction process and can be converted to the active forms, Me-Cbl and Ado-Cbl, following ingestion [3,4].

For human beings, VitB12 is not endogenously synthesized and is a cofactor for vital biochemical reactions [4,16,17]. In the cytoplasm, VitB12, in the form of Me-Cbl, is a methyl group donor and is involved in the synthesis of methionine from HCy and folic acid (FA) (a.k.a. VitB9) performed by methionine synthase (MTR); whereas, in mitochondria, VitB12, as Ado-Cbl, is a co-factor for L-methylmalonyl-CoA mutase (MUT), and participates in the synthesis of succinyl-CoA from L-methylmalonyl-CoA and biotin [3,4].

The uptake of VitB12 to specific cellular sites and its conversion to active forms are a multi-step process. Thus, alterations in VitB12 metabolism may be determined not only by dietary deficiencies or by impairments in the MTR or MUT (enzymes employing VitB12 as a co-factor) but also by other factors involved in its absorption. It is crucial to acknowledge that the clinical presentations resulting from metabolic issues associated with the absorption or processing of VitB12 may exhibit partial overlap with VitB12 deficiency. Hence, a cautious approach is necessary when interpreting the findings. Here, a brief description of the uptake mechanism is first provided, elucidating the factors that can lead to VitB12 malabsorption. A detailed exploration of the biochemical mechanisms underlying metabolic imbalances resulting from VitB12 deficiency, malabsorption, or inborn defects associated with VitB12 metabolism is then undertaken.

### 2.1. The Mechanism of Vitamin B12 Absorption

**Figure 1:**
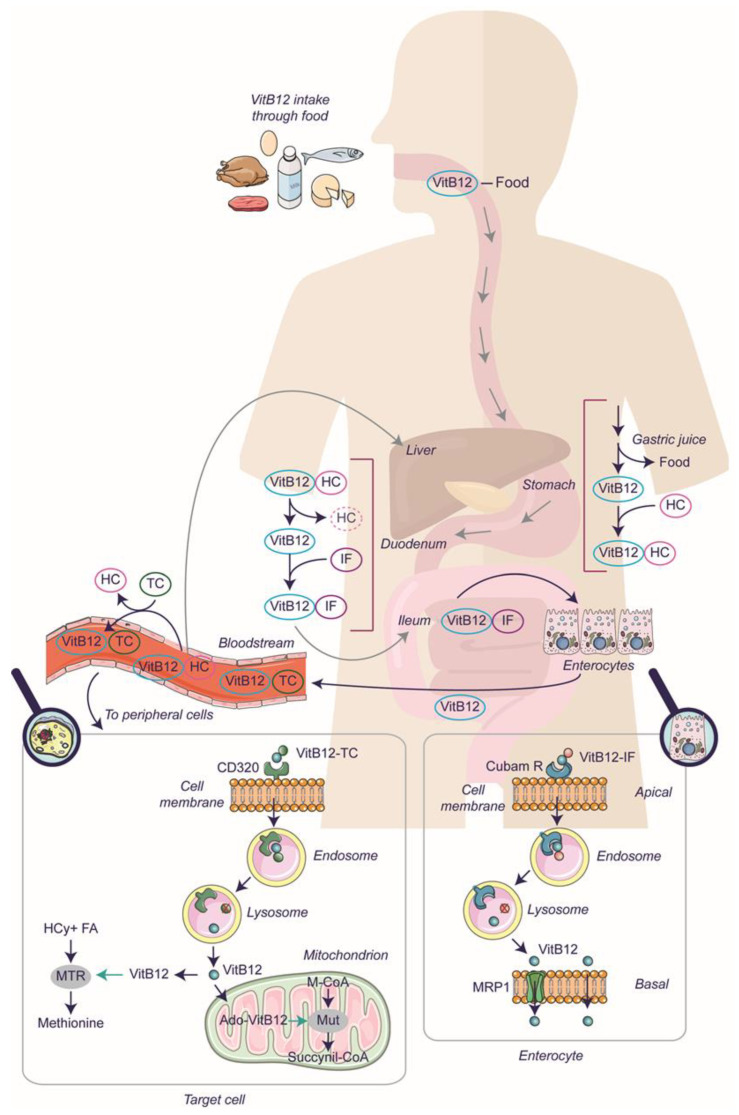
The diagram illustrates the key mechanisms outlined in the text regarding the absorption, storage, and intracellular metabolism of Vitamin B12 in human beings.

In humans, the uptake of VitB12 is a multi-step process (Figure 1), starting in the stomach with the release of VitB12 from food thanks to gastric juices. The free VitB12 binds to Haptocorrin (HC) (also named R-protein or Transcobalamin I and encoded by the TCN1), which is secreted by the oral mucosa. The HC protein has a glycosylated structure conferring resistance to low pH, thereby protecting VitB12 from harsh gastric conditions [18]. The VitB12-HC complex then enters the intestine and undergoes degradation in the duodenum. Pancreatic proteases can degrade the HC, and the resulting free VitB12 associates with Intrinsic Factor (IF) to form a complex. In the ileum, the VitB12-IF is specifically recognized by the cubam receptor on the apical surface of enterocytes. Such binding triggers receptor-mediated endocytosis, resulting in the internalization of the VitB12-IF complex. Subsequently, the complex is transported to lysosomes for further processing, and the cubam receptor is addressed to the plasma membrane to be recycled [19]. Cubam is a multifaceted complex that has not been comprehensively elucidated. It is comprised of two essential components: cubilin (CUB), encoded by the CUBN gene, and amnionless (AMN), encoded by AMN. However, additional proteins may contribute to the stability and function of this complex, including megalin (MAG), encoded by the LRP-2 gene, and receptor-associated protein (RAP), which interacts with specific components of the cubam complex [20,21]. Once the VitB12-IF complex reaches the lysosomes, IF undergoes degradation, releasing free VitB12, which is then actively transported into the cytoplasm with the help of two transmembrane proteins encoded by the LMBRD1 and ABCD4 genes [22]. In the cytoplasm, free VitB12 is conveyed through the basolateral side of enterocytes via active transport (mediated by multi-specific membrane transporter, multidrug-resistant protein 1, MRP1/ABCC1) or passive transport to the blood flow [23]. In the blood, VitB12 can bind with varying degrees of affinity to the known carriers, Transcobalamin (TC) (previously named Transcobalamin II and encoded by the TCN2 gene) and HC. VitB12 bound to TC (forming holotranscobalamin) represents the circulating bioavailable form of VitB12, and is absorbed by the peripheral cells via endocytosis mediated by the receptor CD320 (also known as Transcobalamin II Receptor) [23]. Although not precisely described, a mechanism similar to what occurs in enterocytes takes place in peripheral target cells. Following receptor-mediated endocytosis, within the lysosome, TC is degraded, and the CD320 receptor is recycled to the plasma membrane. In this manner, VitB12, no longer complexed to a protein, can enter the cytoplasm via lysosomal transporters for utilization by specific enzymes (MTR and MUT) and may even be exported [20,21].

Interestingly, about 80% of VitB12 (and inactive VitB12 analogues) is bound to HC, forming holohaptocorrin (VitB12-HC). This complex has a dual function: (i) VitB12-HC conveys VitB12 to the liver, where it undergoes uptake through endocytosis mediated by Asialoglycoprotein receptor (ASGPR); (ii) VitB12 from VitB12-HC can be transferred to TC (which has a higher affinity for VitB12). Thus, VitB12-HC is not only involved in the formation of the hepatic stock of VitB12 but also constitutes a reserve of circulating VitB12. It must be added that a small percentage of VitB12 is available in the blood flow in free form, and hepatocytes, in addition to having the ASGPR receptor, also express CD320, drawing a finely tuned reservoir system yet to be elucidated [24,25,26].

### 2.2. VitB12 Deficiency: The Cause and the Need for Supplementation

The transport of VitB12 to specific cellular locations and its conversion into active forms involve multiple steps, and a disruption in any one of these reactions can impact not only the lack of substrates and inborn errors linked to vitamin uptake/metabolism but also result in VitB12 deficiency. This deficiency can also be the result of autoimmune diseases, malabsorption following inflammatory diseases, and resection of part of the gastrointestinal apparatus [27,28]. For example, considering the formation of the complex VitB12-IF, besides mutations in the gene encoding for IF, several conditions can avoid the accomplishment of this step. In the autoimmune disease pernicious anemia, an anti-intrinsic factor antibody is produced that determines the non-absorption of VitB12 at the terminal ileum, thus making VitB12 not available. Gastric bypass surgery eliminates the IF-producing cells (parietal cells) in the digestion pathway, thus making IF not available. On the other hand, even when IF is available in the correct form, damage in the ileum, including surgical resection due to Crohn’s disease, inflammation from celiac disease, or infection with parasites (*Diphyllobothrium latum* and *Giardia lamblia*), can affect VitB12-IF uptake. Furthermore, evidence suggests a two-way balance of microbiota and VitB12 absorption: microbial intestinal flora is made up of bacteria that can synthesize VitB12 *(Pseudomonas* spp. and *Klebsiella* spp.), transform VitB12 into analogues, or consume VitB12. Furthermore, VitB12 and its analogues (globally known as corrinoids) have a role in bacterial gene modulation, thus suggesting that the levels of the above-mentioned molecules could modulate the microbiota balance [29]. Additionally, distinct endoscopic observations and variations in gastritis are frequently associated with the presence or absence of B12 deficiency [30], and there is a reported positive correlation between levels of VitB12 and Cubam R expression in duodenal mucosa [31]. This suggests that the lack of VitB12 may alter intestinal epithelium, although establishing this link is challenging due to the bidirectional influence between deficient epithelium and VitB12 malabsorption. Recent studies employing multi-omic approaches have shown that VitB12 deficiency disrupts the transcriptional and metabolic programming of ileal epithelial cells. This disruption reduces epithelial mitochondrial respiration and hampers carnitine shuttling, making the epithelial barrier more vulnerable to Salmonella Typhimurium [32,33]. Moreover, as later discussed, there exists a reciprocal relationship between microbiota and VitB12, and alterations in the microbiota may affect the intestinal epithelium [32,33,34].

It is to be noted that, although gut microbes can produce VitB12, they do not provide significant sources of cobalamin to humans for several reasons, including the fact that the total cobalamin found in the feces is only 2% of total need, and is produced mainly in the colon, which is downstream of the ileum, thus excluding the possibility of absorbtion [29].

The VitB12 stocked in the liver can be made available by reabsorption of the biliary cobalamin [24,25,35]. In humans, VitB12′s daily requirement is 1–4 μg, and the liver stores roughly 2–5 mg. Accordingly, VitB12 deficiency symptoms typically develop within 3–5 years of the beginning of the malabsorption, thus making the disease chronic and impairments not reversible in some cases [11].

Deficiency of VitB12, various inborn errors that can affect the absorption (such as IF deficiency and Imerslund–Gräsbeck syndrome), the transport (transcobalamin deficiency), and the intracellular metabolism of VitB12 (combined methylmalonic acidemia and homocystinuria depending on the genes involved), rely on a specific requirement of supplementation/treatment with VitB12 that allow patients to survive and, in some cases, improve neurological impairments. Moreover, medical evidence also suggests a beneficial role of high VitB12 dosage on some of the neuronal impairments [12].

### 2.3. Metabolic Decompensation after VitB12 Deficiency

**Figure 2:**
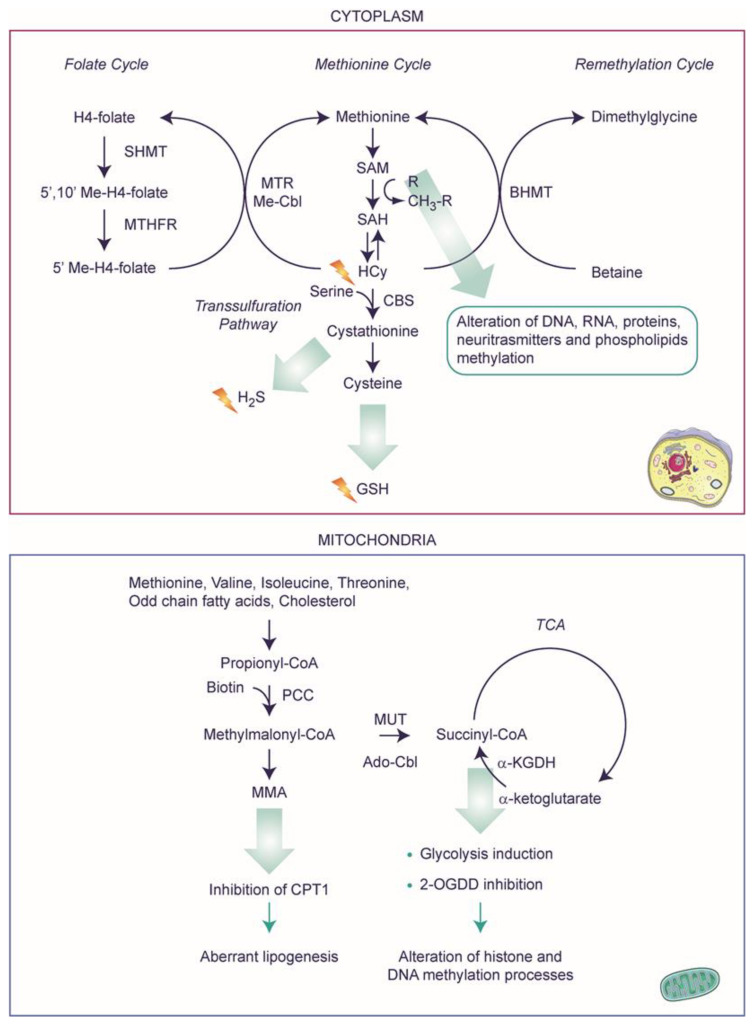
The lack of Vitamin B12 notably impacts MTR or MUT, enzymes reliant on it as a cofactor. This deficiency not only disrupts the biosynthesis of crucial compounds but also triggers the accumulation of reaction intermediates that are potentially toxic at increased concentrations, leading to metabolic decompensation. Lightning bolt icons indicate the metabolic steps that might indirectly contribute to oxidative stress. The light green arrows highlight further biochemical implications resulting from Vitamin B12 deficiency, potentially influencing molecular and cellular processes.

The deficiency of VitB12, or more specifically, the alterations in MTR or MUT (for which VitB12 serves as a cofactor), not only avoids the biosynthesis of reaction intermediates but also determines the accumulation of substrates that are toxic at higher concentrations (Figure 2). In particular, alterations in MTR activity can block the methionine cycle and result in the non-synthesis of methionine and hyperhomocysteinemia (i.e., a high level of toxic HCy). However, through a parallel pathway, HCy can be converted into methionine and cystathionine with the involvement of betaine-homocysteine S-methyltransferase (BHMT) and cystathionine-beta-synthase (CBS), respectively. This results in a methionine pool that can be further converted into S-adenosyl-L-methionine (SAM) [36,37]. In mitochondria, VitB12-related alterations can alter the lipid metabolic pathways and reduce succinyl levels, thereby modifying the mitochondrial energy production balance. Note that some amino acids (namely methionine, threonine, isoleucine, and valine), cholesterol, and beta-oxidation of odd-numbered carbon chain fatty acids can lead to the synthesis of propionyl-CoA; the latter can be carboxylated into methylmalonyl-CoA by propionyl-CoA carboxylase, a biotin-dependent enzyme. Methylmalonyl-CoA, the substrate of L-methylmalonyl-CoA mutase, is then converted into succinyl-CoA in a VitB12-dependent reaction as mentioned above. Alterations in L-methylmalonyl-CoA mutase block the production of succinyl-CoA (although it can be alternatively obtained by the oxidative decarboxylation of alpha-ketoglutarate) [38], resulting in increased levels of MMA, methylmalonyl-CoA, and propionic acid. Furthermore, methylmalonyl-CoA inhibits carnitine palmitoyltransferase-1 (CPT1), which is responsible for converting long-chain acyl-CoAs to long-chain acyl-carnitines, the form of fatty acid that can be imported into the mitochondria. This could lead to a decrease in fatty acid oxidation and aberrant lipogenesis [35,39]. The independent role of VitB12 on lipid metabolism, a key risk factor for cardiometabolic disorders, has not been explored to a larger extent [35].

To provide a comprehensive picture, it is important to note that both forms of VitB12, natural OH-Cbl and synthetic CN-Cbl, undergo a series of biosynthetic modifications that lead to their conversion into the active forms, Me-Cbl and Ado-Cbl. This conversion process involves several enzymes, although not all of them have been fully characterized. Currently, nine enzymes have been described, the alteration of which can block the production or utilization of Me-Cbl, Ado-Cbl, or both cofactors: lipocalin-1 interacting membrane receptor domain-containing protein 1 (LMBD1), ATP-binding cassette subfamily D member 4 (ABCD4), methylmalonic aciduria type C and homocystinuria (MMACHC), methylmalonic aciduria type D and homocystinuria (MMADHC), methylmalonic aciduria type A (MMAA), ATP-dependent cob(I)alamin adenosyltransferase (ATR), methylmalonyl-CoA mutase (MUT), methionine synthase reductase (MSR) and methionine synthase (MTR) encoded by *LMBRD1*, *ABCD4*, *MMACHC*, *MMADHC*, *MMAA*, *MMAB*, *MUT*, *MTRR*, and *MTR*, respectively. Several mutations have been found to be associated with the mentioned genes, forming nine complementation groups. In particular, mutations in *LMBRD1*, *ABCD4*, and *MMACHC* genes (corresponding to cblF, cblJ, and cblC complementation groups) are associated with combined methylmalonic aciduria and homocystinuria. Mutations in *MMAA*, *MMAB*, and *MUT* (corresponding to cblA, cblB, and mut complementation groups) are associated with isolated methylmalonic aciduria, while mutations in *MTRR* and *MTR* (corresponding to cblE and cblG complementation groups) are associated with isolated homocystinuria. Mutations in *MMADHC* (belonging to the cblD complementation group) can be associated with all three clinical phenotypes [40].

### 2.4. A Speculative Scenario of VitB12 Deficiency

Though lacking precise pathological studies, considering our current understanding of physiological pathways, it is possible to speculate on additional biochemical implications of VitB12 deficiency that may affect molecular and cellular processes (Figure 2). In terms of energy metabolism, the insufficiency of VitB12 hampers the proper functioning of the Krebs cycle, as the conversion of methylmalonyl-CoA to succinyl-CoA is impeded. Consequently, cells might rely on aerobic glycolysis rather than respiration for energy production [41,42]. This shift in energy production may be exacerbated by the reduced cellular content of the succinyl-CoA product, specifically succinate, whose low levels are associated with the induction of the transcription of glycolytic genes [43,44]. Furthermore, decreased succinate levels can limit its inhibitory effect on 2-oxoglutarate-dependent dioxygenase (2-OGDD) enzymes, which include histone (JMJD) and DNA (TET) demethylases, thereby influencing the histone and DNA methylation processes. This phenomenon could be further exacerbated by the depletion of the methyl donor S-adenosylmethionine (SAM) pool, as a consequence of VitB12 deficiency, a universal methyl donor needed for the methylation of DNA, RNA, proteins, neurotransmitters, and phospholipids. Indeed, the lack of methionine following VitB12 deficiency can lead to SAM depletion. Additionally, the methylation reactions utilizing SAM produce S-adenosylhomocysteine (SAH). Normally, SAH undergoes hydrolysis, aiding in the recycling of adenosine and homocysteine. However, the accumulation of homocysteine (HCy) resulting from VitB12 deficiency causes an excessive build-up of SAH, which then inhibits methyltransferases. Consequently, these combined phenomena lead to significant molecular changes, including epigenetic modifications of DNA and histone proteins, ultimately influencing gene expression [45].

A deficiency in VitB12 is known also to be associated with an increase in oxidative stress. While a reciprocal relationship between oxidative stress and inflammation has been observed in the context of VitB12 deficiency (see below), the exact underlying mechanism of oxidative stress generation remains poorly understood [46]. For example, VitB12 possesses scavenging activity, and thus, its absence may directly contribute to an increase in oxidative stress. Additionally, it is believed that an excess of homocysteine (HCy) can undergo auto-oxidation, leading to the production of hydrogen peroxide [46]. Furthermore, HCy can be directed towards the initial synthesis of cysteine, which subsequently leads to the production of glutathione (GSH) (a vital antioxidant molecule). The desulfhydration process of homocysteine by cystathionine-β-synthase (CBS) needs SAM to act as an allosteric activator, and produces cysteine through cystathionine generating hydrogen sulfide (H_2_S), a molecule recognized for its anti-inflammatory and antioxidant effects. A deficiency of VitB12, which in turn leads to a decrease in SAM, can indirectly affect the production of both GSH and H_2_S [47]. Finally, the increased oxidative stress within the cell can alter the oxidation state of cobalamin, subsequently affecting VitB12 metabolism. Indeed, the higher oxidation state of cobalamin inhibits methionine synthase activity, thwarting the conversion of HCy to methionine. This intricate scenario emphasizes how a deficiency in VitB12 exerts far-reaching effects within the cell, extending beyond mere biochemical alterations.

## 3. Vitamin B12 Deficiency: What Can We Learn from Experimental Models?

**Figure 3:**
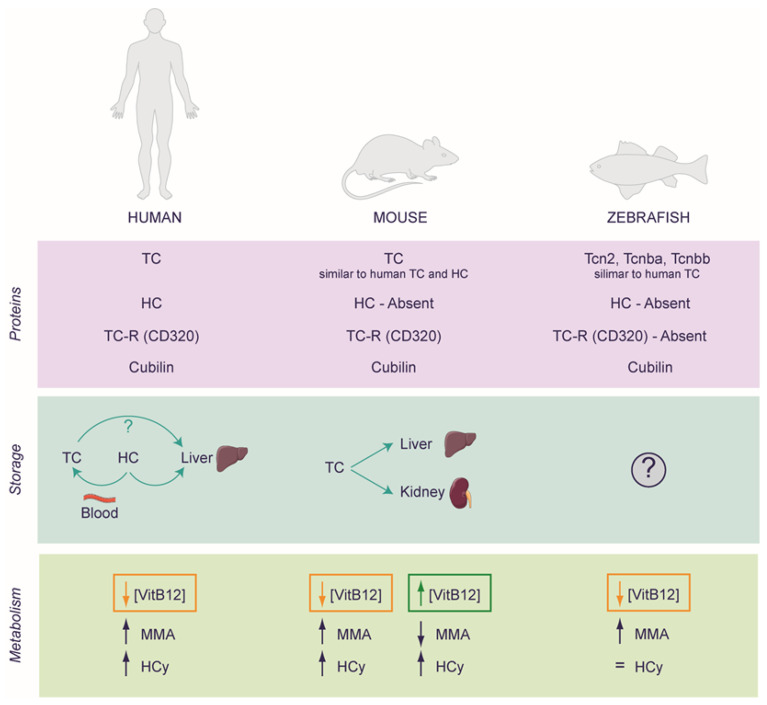
Divergent metabolic processes and uptake mechanisms of Vitamin B12 across human, mouse, and zebrafish. The question mark indicates that the mechanism has been poorly characterized. The arrows in the ‘metabolism’ part of the figure indicate an increase (if pointing upwards) or a decrease (if pointing downwards) in the metabolite or vitamin B12, while the equal sign indicates no change.

This review draws upon a diverse array of data and results sourced from various models, encompassing cellular origins in both human and murine contexts, alongside animal models involving mice and zebrafish (*Danio rerio*). It is noteworthy that these organisms may display differences in the metabolic processes and uptake mechanisms of VitB12 that may not be conserved in the species considered. Therefore, observations collected in one model may not be extended to another one, requiring further elucidation to provide a better understanding (Figure 3 and Table 1). Moreover, inducing a state of VitB12 deficiency proves to be a challenging endeavor, and the other pivotal facet warranting scrutiny pertains to the administration of the optimal VitB12 dosage. Within this section, we will thoroughly explore these intricacies, which are strongly linked to the experimental challenges within the specific scope of VitB12 research.

### 3.1. VitB12 Deficiency and Supplementation in Experimental Models: Far from Simple

One of the main challenges in investigating VitB12 deficiency or potential benefits of supplementation is in setting the state of deficiency or the appropriate dosage of supplements. Regarding human cell cultures, there are two major issues. The most common cell culture media lacks VitB12 (MEM, D-MEM, L-15), and other media contain concentrations between 0.28 to 1 μmol/L (M-199, HAM F-10, HAM F-12, RPMI-1640, DMEM/HAM F12), while treated fetal bovine serum has VitB12 in trace [55]. Note that in human serum, the levels of ViB12 may vary slightly in different reports due to several influencing factors, including regional differences, individual health conditions, and testing methodologies. For instance, Ermens and co-workers reported that values between 250 and 850 pM are considered normal for healthy individuals [56]. This aligns with Carmel, which indicates low serum levels of VitB12 for values below 148 pM, low-normal between 148–258 pM, and normal or high for values above 258 pM [57]. Thus, when media containing VitB12 are employed, its concentration can be 300 to 1200 times higher than that found in human serum. On the contrary, cells growing in a medium lacking VitB12 can potentially exhibit VitB12 deficiency, alongside possible deficiencies in other essential nutrients. Moreover, achieving a state of VitB12 deficiency sufficient for detecting key biochemical markers, such as elevated levels of MMA or HCy, necessitates a significant amount of time. Unfortunately, this extended period often leads to the demise of the cell culture due to the starvation process. Conversely, when deficiency is achieved by treating cell cultures with an anti-VitB12, successful intracellular localization of the inhibitor hinges on its endocytosis. This process depends on the availability of a pool of Transcobalamin (TC), which leads to the formation of the VitB12-TC complex, followed by the occurrence of CD320-mediated endocytosis (reviewed by [23,58]). TC is generally found in the serum added to the medium (although the protein composition of serum is not standardized). Thus, the serum is a potential source of TC but also contains VitB12 in trace [55]. Consequently, achieving complete inhibition, total VitB12 deficiency, or a complete supplementation setup becomes complex. These issues have pushed researchers to explore genetically engineered models by targeting genes integral to the uptake or metabolism of VitB12, thereby aiming to establish systems that recapitulate VitB12 deficiency (see below). Indeed, through genetic manipulation of genes related to the absorption or metabolism of VitB12, a variety of animal models have been generated. These models can mimic VitB12 deficiency and enhance our comprehension concerning the biological process. Lastly, further consideration to be considered is the optimal dosage of VitB12 employed within the experimental designs to explore its beneficial effects. Whenever feasible, in this review, the dose is reported aiming to ascertain their comparability to physiological levels and/or current treatments employed in human beings (see below).

Numerous studies focusing on neuronal function (including part of the data reported in this work), related pathologies, and VitB12 utilize SH-SY5Y human neuroblastoma cell line. These cells exhibit several characteristics of mature neurons, making them valuable for investigating various aspects of human neuronal biology. There are several important considerations to be made regarding this cellular model. Firstly, it should be noted that the SH-SY5Y cell line, despite its widespread usage due to its easy maintenance, is originally derived from a human brain tumor. Furthermore, researchers often utilize the SH-SY5Y cells in their undifferentiated state, as these cells already exhibit neurogenic markers even during their proliferative phase. However, they can also be differentiated into neuron-like cells, characterized by their triangular cell bodies and the formation of neurite outgrowth processes. The most employed methods of differentiation involve the use of retinoic acid alone, retinoic acid in combination with brain-derived neurotrophic factor (BDNF), or retinoic acid in combination with phorbol 12-myristate 13-acetate (PMA). It is essential to recognize that the specific differentiation agent employed can significantly affect the global biology of the cells and raise certain implications that need to be considered when interpreting experimental results [59].

The complexities of VitB12 biology cannot be entirely replicated using 2D cell cultures alone. Nonetheless, these cultures provide critical insights into cellular and molecular mechanisms, forming the foundation for further exploration within organismal models.

### 3.2. Rodents as a Model for Studying VitB12 Deficiency

The system of VitB12 transporters exhibits slight differences between mice and humans. Mice lack HC, and TC shares characteristics of both human TC and HC, as it recognizes both VitB12 and analogues. Furthermore, under normal conditions, about 10% of circulating TC in humans is saturated with VitB12 compared to 50% in mice. The difference in the biology of VitB12 is evident considering the kidney’s role in rodents. In mice, the kidney acts as a VitB12 reservoir, accumulating the vitamin during loading and releasing it during deficiency, indicating a certain difference in the uptake and storage of VitB12 in this model [48]. Indeed, as mentioned previously, in humans, the organ responsible for storage is the liver and partially the blood, although the kidney plays a crucial role in the reabsorption of TC [48,60]. Based on these observations, Lildballe and co-workers, to investigate the effect of an excess or a deficiency of VitB12, designed a mouse model to continuously saturate the endogenous mouse TC with VitB12 or with the anti-VitB12 cobinamide (Cbi) using osmotic minipumps. The researchers achieved the maximally loaded transport system as all circulating TC remained saturated, and the excess of VitB12 or Cbi was excreted in the urine. The dose was 42 nmol/24 h and 102 nmol/24 h of VitB12 and Cbi, respectively. It is important to note that, on average, mice have approximately 58.5 mL of blood per kg of body weight. Therefore, a 25 g mouse would have a total blood volume of approximately 1.46 mL (58.5 mL/kg × 0.025 kg). Thus, the resulting concentrations of VitB12 and Cbi in the blood could theoretically reach 28.77 μM and 69.86 μM, respectively, per day. This corresponds to approximately 34,000 times the normal content of VitB12 in humans and about 82,000 times the amount of an antivitamin equivalent to normal values of VitB12 (considering the serum concentration of 850 pM as a reference [56]). When mice were subjected to elevated doses of VitB12, their plasma levels of VitB12 increased. Simultaneously, a decrease in the expression of Methylenetetrahydrofolate reductase (MTHFR) was observed in their kidney tissues. MTHFR is an enzyme essential for converting 5,10-methylenetetrahydrofolate into 5-methyltetrahydrofolate, which, in turn, acts as a methyl donor for the methylation of homocysteine to methionine, a process that depends on the presence of VitB12. Surprisingly, following a high dose of VitB12, although the level of MMA decreased, HCy levels increased, thereby suggesting that both insufficient and excessive VitB12 levels could negatively impact the HCy metabolism in a mouse model. Moreover, the excess VitB12 is accumulated primarily in the kidney, although the liver doubled its VitB12 uptake. Furthermore, the study revealed downregulation of TC and CD320 (i.e., the TC receptor) in the salivary gland, following a high-dose VitB12 treatment. Prolonged treatment with inactive VitB12 analogues like Cbi could eventually lead to VitB12 deficiency, although it does not influence the markers of VitB12 metabolism in mice (the red blood cell counts and the levels of MMA and HCy were unchanged). However, these markers are not well understood in mice, as mice with alterations in some proteins involved in the metabolism of VitB12, showing reduced tissue VitB12 levels, do not exhibit haematic alterations [61]. Interestingly, in both treated groups (high dose of VitB12 and Cbi), the white blood cell count increased, reaching the highest level in the VitB12-treated mice group. A comparable response has been observed in humans with excessive VitB12 load, where VitB12 was found to act as a cellular modulator in the immune response system [62]. Similar results were obtained using another antivitamin inhibitor, Coβ-4-ethylphenyl-cob(III)alamin, EtPhCbl [49]. In a study conducted by Mutti and coworkers, mice were treated with EtPhCbl, which binds to TC and can induce cellular VitB12 deficiency. The animals were administered 3.5 nmol/24 h of EtPhCbl, 3.5 nmol/24 h of VitB12 (CN-Cbl), or NaCl (control group) via osmotic mini-pumps over a period of four weeks. Thus, the concentration of VitB12 or EtPhCbl per day could have theoretically reached about 2.40 μM, roughly 2800 times the normal content of VitB12 in humans (considering the serum concentration of 850 pM as a reference [56]). The researchers analyzed plasma, urine, liver, spleen, submaxillary glands, and spinal cord for VitB12 levels and markers of VitB12 deficiency, including the levels of MMA and HCy. Animals treated with EtPhCbl showed elevated plasma MMA levels compared to controls and CN-Cbl-treated animals. A similar pattern was observed for HCy. It should be noted that the observed organs exhibit varying absorption capabilities for both VitB12 and EtPhCbl. Particularly, the spinal cord appears to be resistant to both treatments, suggesting the influence of biological barriers on VitB12 absorption; however, regarding the experiment of Mutti and colleagues, a global deficiency of VitB12 is observable when animals were treated with EtPhCbl [49]. Interestingly, mice with autoimmune encephalomyelitis (EAE) show both a reduction of CD320 and a lower level of VitB12 in the spinal cord, and these levels of VitB12 worsen when the animals are subjected to a VitB12-deficient chow diet [63]. These data, which appear to be in apparent discord, might imply a complex regulatory system for VitB12 levels in the spinal cord that remains unclear and possibly linked to the progression of the disease, particularly in experimental models.

Despite the mentioned differences in mice and humans regarding VitB12 uptake, observations from the human SH-SY5Y cell model and the mouse brain show a similar intracellular trafficking of VitB12. SH-SY5Y cells exhibit a distribution of VitB12 in the lysosomes, mitochondria, and cytosol, accounting for approximately 6%, 14%, and 80%, respectively. Similarly, in the mouse brain organelles, the relative distribution of VitB12 was approximately 12% in lysosomes, 15% in mitochondria, and 73% in cytosol [64,65].

### 3.3. Zebrafish as a Model for Studying VitB12 Deficiency

Another widely utilized experimental model for studying the role of VitB12 at the organism level is the zebrafish. The zebrafish possesses more cobalamin transport proteins, namely Tcn2, Tcn-beta-a (Tcnba), and Tcn-beta-b (Tcnbb). Interestingly, Tcn2, a TC homolog, possesses both the α-domain and β-domain, which work together to form a binding cleft, facilitating the attachment of cobalamin; in contrast, Tcnba and Tcnbb lack the α-domain, however, they exhibit a remarkably high affinity for cobalamin, closely resembling Tcn2. Furthermore, the zebrafish lacks a homolog of the TC receptor, CD320, although a homolog of cubilin, involved in the receptor-mediated endocytosis of VitB12-IF in humans, has been identified. In humans, TC recognition by CD320 is driven by contact with only the α-domain, while the binding kinetics of IF involve contact with the α-domain and β-domain of its receptor, cubilin. This indicates that zebrafish may employ an alternative receptor or pathway for cobalamin uptake into cells [66]. With regard to the VitB12 intracellular metabolism, Sloan and coworkers used an in silico approach to identify potential zebrafish orthologs of human genes. Many of these orthologs were found to be highly conserved, including *mmachc*, the zebrafish ortholog of MMACHC [54]. In humans, the MMACHC gene encodes for CblC, a protein involved in the final step of Ado-Cbl and Me-Cbl synthesis. Mutations in MMACHC are associated with methylmalonic aciduria and homocystinuria, cblC type (cblC) (MIM 277400), the most prevalent inborn error in intracellular VitB12 metabolism [67]. This condition is characterized by a complex array of symptoms, including metabolic decompensation related to VitB12, neurological abnormalities, ocular deficits, and craniofacial dysmorphia [68,69]. For instance, it was observed that mmachc shares 51% sequence identity with the human protein but differs at the C-terminus, resulting in a shorter protein (250 amino acids in zebrafish versus 282 amino acids in humans). These findings indicate a certain degree of similarity in intracellular VitB12 metabolism in the zebrafish compared to humans.

In the zebrafish, a mmachc morphant mutant exhibited a severe embryonic phenotype characterized by growth impairment, developmental delay, axis development abnormalities, impaired swimming, pericardial effusion, and brain edema. On the contrary, the germinal mutant using zinc finger nucleases (ZFNs) approach to target exon 2 of mmachc, in order to reproduce a similar common human pathogenic variant (c.271dupA p.Arg91Lysfs*14), led to different outcomes. Allele engineering determined the frameshift mutations and led to the nonsense-mediated decay of mmachc mRNA. Interestingly, unlike the mmachc morphants, ZFN-generated fish models survived the embryonic period and appeared at expected Mendelian ratios during the larval period. However, at 35 days post-fertilization (dpf), more than 90% of the homozygous mutants died, resulting in a skewed genotype distribution, and no homozygous mutants survived past 42 dpf [54]. The notable disparity observed between morphant mutants and germline mutants can be attributed to the different methods employed in generating these models. Morphant mutants are created using morpholinos or simRNA to target the mRNA of embryos, including maternal mRNA. On the other hand, germline mutants are generated using genome editing techniques, such as ZFNs, to introduce genetic modifications into zebrafish embryos at the one-cell stage or early developmental stages. Using the ZFNs approach, during this early stage, the zygotic genome is not yet active, and the embryo heavily relies on maternal mRNA for essential functions. Consequently, the presence of maternal mRNA can support the early stage of development even in mutated animals, allowing embryonic development to proceed [54]. Similar outcomes have been observed in mouse models as well [50].

The mmachc mutant zebrafish produced by Sloan and co-workers exhibited symptoms similar to human patients with cblC deficiency, including metabolic perturbations such as elevated levels of MMA, as well as increased oxidative stress and mild craniofacial dysmorphia [54]. The observation of mild craniofacial dysmorphia phenotype in zebrafish opens up new questions regarding the role of mmachc. Indeed, craniofacial dysmorphia is observed also in mice carrying the *Mmachc* mutation and is associated with alteration in HCy levels that can be detected in the model [37,50]. However, zebrafish lacking the expression of mmachc show mild craniofacial dysmorphia and no alterations in HCy levels [53], suggesting the outcome is not dependent on the alteration of metabolism but possibly due to specific (and unknown) activities of mmachc unrelated to VitB12 metabolism as a coenzyme. A similar conclusion can be made considering eye disease. Mutant zebrafish exhibited retinal degeneration and dysregulation of genes related to phototransduction and metabolism [54], similar to cblC-associated eye disease in humans, generally attributed to metabolic decompensation, specifically, hyperhomocysteinemia [70]. These observations provide a much better understanding that the previously observed defects may not be linked to VitB12 metabolic alterations but could be possibly due to its additional roles, extending beyond the metabolic functions of the proteins involved in the biochemical pathway. For instance, considering the occurrence of maculopathy and progressive retinal degeneration among patients with clbC and, to some extent, patients with cblD, interestingly, such conditions do not manifest in patients with mutations in the other genes associated with VitB12 metabolism [50]. Furthermore, the physical interaction between MMADHC and MMACHC has been identified, and their interaction has been hypothesized to mediate functions not directly related to VitB12 metabolism [71,72]. These less-known functions could partially explain the excellent clinical presentation of the patient who underwent intrauterine treatment and shows only mild ophthalmic issues [13], and the cases with late-onset symptoms that have been documented in individuals harboring mutations in the genes involved with VitB12 metabolism. Interestingly, patients with late-onset symptoms, despite carrying a mutation, develop symptoms only during their adolescence or adulthood [73,74,75,76,77,78]. This suggests the existence of mechanisms that could either delay the metabolic onset of the disease or, conversely, trigger it, and also suggests the possibility that some impairments could not be due to metabolic unbalance. Therefore, providing a definitive answer proves challenging, primarily due to the wide array of mutations (identical alterations on both alleles, different mutations for each allele, or even classic heterozygosity). Moreover, as mentioned before, the complexity deepens with the realization that these proteins are possibly engaged in functions extending beyond VitB12 metabolism. This additional role in function brings yet another facet of intricacy to the overall picture.

A concluding point worth emphasizing is that, although the models discussed here do not precisely mirror human physiology, they provide valuable insights into the potential outcomes of VitB12 deficiency, whether induced by dietary insufficiency or genetic manipulation, and the possible improvement of the current therapies. For example, individuals suffering from VitB12 deficiency are typically supplemented with OH-Cbl or CN-Cbl [8]. On the other hand, patients with cblC-related issues receive treatment involving parenteral OH-Cbl, betaine, and other supplements aimed at enhancing biochemical markers. However, the dosage of these treatments primarily depends on the case reports and medical practitioners’ preferences, taking into account the patients’ responses to therapeutic interventions, which can significantly vary among individuals [68]. Using the zebrafish model, the researchers observed that Me-Cbl treatment proved to be the most effective for improving the growth parameters in the cblC zebrafish, despite no significant alterations in the measured metabolites. This suggests that further exploration of Me-Cbl treatment in cblC patients could be considered; however, the treatment might require a combination with OH-Cbl, as Me-Cbl alone revealed only a minor decrease in the MMA levels in the zebrafish model [54]. Finally, it is worth noting that while organisms can offer more comprehensive insights into the biology of VitB12 compared to cells, which provide a more focused and simpler means of investigation into a specific cell type, various considerations need to be applied when using animal models. For example, although the zebrafish presents opportunities to gather information on the embryonic development and metabolic functions of VitB12 due to the conservation of many orthologs directly involved in metabolism, such as mmachc, it cannot be employed to investigate absorption-related issues in human beings due to disparities in this pathway between humans and zebrafish

## 4. Vitamin B12 Deficiency and Nervous System: Still Lacking Crucial Knowledge

**Figure 4:**
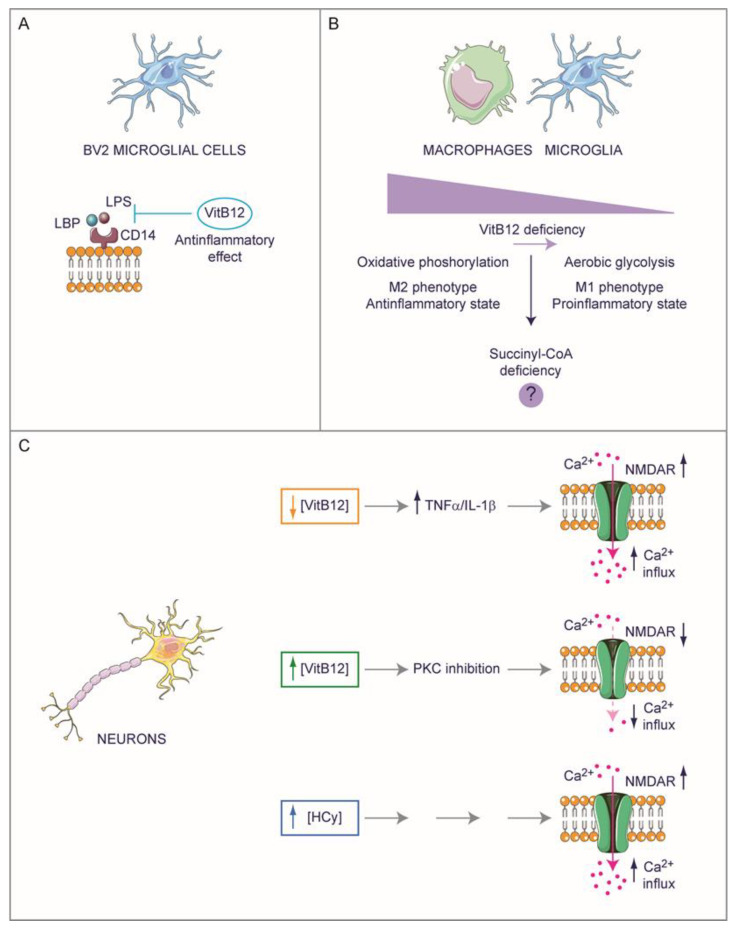
Vitamin B12’s functions as an anti-inflammatory agent by binding to CD14 (**A**), modulating potential energetic metabolism (**B**), and mediating NMDA receptor-linked excitotoxicity (**C**).

**Figure 5:**
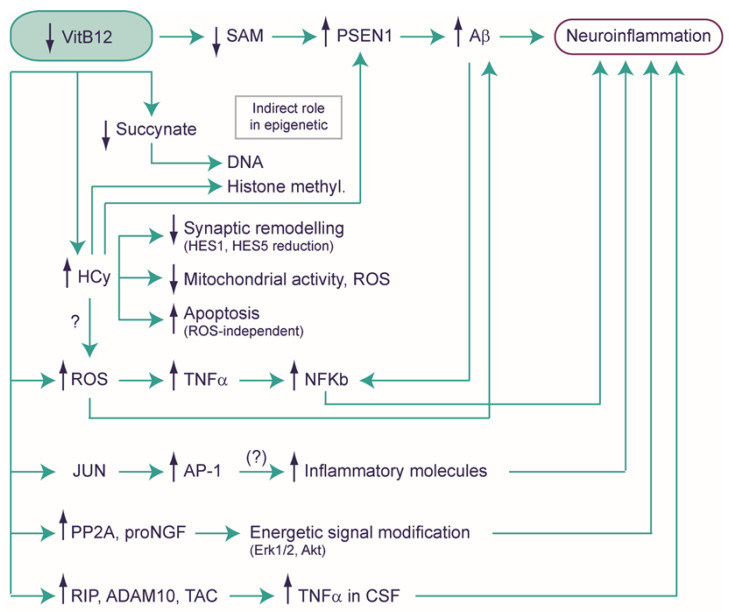
This outline explores the molecular and cellular changes in the nervous system during Vitamin B12 deficiency, potentially leading to neuroinflammation. It emphasizes that metabolic decompensation is just one facet of this intricate scenario. The question mark indicates that the mechanism has been poorly characterized. The dark blue arrows indicate an increase (if pointing upwards) or a decrease (if pointing downwards) of the compound or of the process.

The symptomatology associated with VitB12 deficiency clearly highlights how the lack of this essential micronutrient can result in impairments related to the nervous system. As mentioned above, VitB12 deficiency in children and infants, stemming from malnutrition or the strict vegan diet of lactating mothers, can impact their development, resulting in various cognitive and movement disorders as well as developmental delays [8]. This vulnerability is also evident in children born to pregnant women, who are at an increased risk of VitB12 deficiency due to factors such as being a vegetarian or vegan, having Crohn’s or celiac disease, or having undergone gastric bypass surgery [79,80]. In adults, VitB12 deficiency can give rise to numerous neurological disorders, such as numbness and tingling in limbs, depression, confusion, dementia, and optic neuropathy [5,6,7]. Furthermore, VitB12 deficiency is associated with cognitive functions, and while not entirely confirmed, some researchers suggest it can present as a risk factor for Alzheimer’s disease (AD) [81]. It is also linked to many other neurological disorders, such as Wernicke’s encephalopathy and subacute combined degeneration of the spinal cord and peripheral neuropathy [81]. These pieces of evidence are some examples that have spurred researchers to investigate potential alterations in the nervous systems, particularly at the biochemical level, shedding light on the intricacies resulting from VitB12 deficiency. However, recent studies have started to reveal that the impacts of VitB12 deficiency extend beyond mere substrate deficiencies or accumulated intermediates (metabolic decompensation), and also encompass more profound cellular transformations, involving modifications at the molecular and cellular levels. In this context, we explore the biochemical shifts and also delve into those of a molecular and cellular nature in the nervous system (Figure 4 and Figure 5). As discussed previously, establishing a system for either VitB12 deprivation or supplementation poses several challenges. Therefore, it is crucial to recognize that many of the concepts reviewed here are based on findings drawn from various sources, often involving speculation extrapolated from knowledge regarding non-pathological biochemical pathways.

### 4.1. Exploring the Potential Role of Vitamin B12 in Neuroinflammation

A recent study has reported that the complex of B vitamins (B1, B2, B3, B5, B6, and B12) can exert anti-inflammatory effects on BV2 microglial cells activated with lipopolysaccharides (LPS), inducing changes in the phenotype profile from M1 toward the M2 microglia type. Through in silico analyses, the authors hypothesized that this effect could be due to the high binding affinity of VitB12 with the CD14 receptor that could inhibit LPS transport and thereby, avoid TLR4 activation (Figure 4A) [82]. It is interesting to note that immune cells like microglia and macrophages shift from oxidative phosphorylation (typical of M2 type phenotype), which corresponds to an inactive state, to aerobic glycolysis (typical of M1 type phenotype), which corresponds to a proinflammatory state [41,42]. This forceful change in metabolism could be due to the deficiency of succinyl-CoA, which is a consequence of VitB12 deficiency, although no substantial data exists (Figure 4B). Many other studies have reported observations that suggest a strong link between neuroinflammation and VitB12 deficiency (Figure 5). For instance, VitB12, by affecting the SAM pool, can modulate the methylation status of the PSEN1 promoter. PSEN1 [83,84,85], a key player in the γ-secretase complex, crucially cleaves amyloid precursor protein (APP) to generate the β-amyloid peptide (Aβ). The latter is a principal constituent of amyloid plaques and is often associated with neuroinflammation [86]. A further link between VitB12 and neuroinflammation is given by the resulting increase in HCy after deficiency; the suppression of dietary VitB12 triggers an elevation of HCy, subsequently intensifying levels of PSEN1, BACE1 (the β-secretase enzyme), APP phosphorylation, and Aβ production within the rodent brain [84,86,87,88,89]. Furthermore, the absence of VitB12 triggers the production of reactive oxygen species (ROS), culminating in an increase in Aβ generation [90,91] and an upregulation of tumor necrosis factor-α (TNF-α) [92,93]. Notably, VitB12 deficiency is linked to increased TNF-α expression and decreased EGF (Epidermal Growth Factor) synthesis within the CNS of rats [94]. This imbalance significantly contributes to myelin damage and vacuolation [95,96]. Importantly, while TNF-α is associated with the inflammatory process, it seems that the myelin-damaging effect observed after VitB12 deficiency operates independently of its pro-inflammatory role. This has been substantiated through studies involving VitB12-deficient rats displaying Spinal Cord Demyelination (SCD) and in the cerebrospinal fluid (CSF) of patients with SCD due to VitB12 deficiency [10]. Subsequent studies have further elucidated the role of VitB12 in supplementation, showing that it can downregulate NF-κB and consequently decrease the level of TNF-α in the peripheral nervous system [93]. Moreover, an increase in the Jun mRNA levels was observed in VitB12-deficient mice, which could potentially lead to an increase in the JUN protein, forming the AP-1 heterodimer alongside FOS [97]. This could potentially contribute to increased neuroinflammation, considering AP-1 to be a pivotal regulator of gene transcription encoding cytokines, chemokines, and other proteins crucial for T-cell recruitment and ROS production [98]. The possible anti-inflammatory role of VitB12 is also suggested by Battaglia and co-workers, by establishing a VitB12-deficient condition in N1E-115 neuroblastoma, a cell line commonly used as a motor-like neuron model [99]. This was achieved by engineering the expression of a modified transcobalamin receptor, transcobalamin-oleosin (TO), which anchors to the intracellular membrane leading to the intracellular sequestration of VitB12. Consequently, the levels of Me-Cbl and SAM are reduced, whereas the MMA and HCy concentration levels increase. Interestingly, in this VitB12-deficiency model, the researchers observed an upregulation of phosphatase 2A (PP2A) and pro-nerve growth factor (proNGF), and these regulatory factors can influence the energetic signaling pathways involving ERK1/2 and Akt. Furthermore, the VitB12 deficiency mimicking conditions lead to an increase in p75NTR-regulated intramembranous proteolysis (RIP), associated with the elevated expression of two TNF-α converting enzymes (TACEs) and secretase enzymes, Adam 10 and Adam 17. These findings align with the observed increase in the TNF-α levels in the cerebrospinal fluid of patients and animal models with VitB12 deficiency, and the higher TNF-α levels may be attributed to the augmented TACE activity [99]. It is important to note that a deficiency in VitB12 in other human neuronal models, such as SK-N-SH and SK-N-BE, leads to a reduction in the expression of the Adam 10 gene [84].

The above-mentioned proinflammatory effect due to VitB12 deficiency could potentially impact neuronal physiology. Proinflammatory cytokines induced by VitB12 deficiency, like TNF-α (but potentially IL-1β, see below), could overactivate the N-methyl-D-aspartate (NMDA) receptors, subsequently causing excessive Ca^2+^ influx that triggers excitotoxicity [100]. In rats, VitB12 can inhibit protein kinase C (PKC) and, in turn, suppress voltage-dependent Ca^2+^ channel activity, decreasing Ca^2+^ influx into synaptosomes [101]. Interestingly, HCy can induce the expression of NMDA receptors and their phosphorylation [102], which could alter NMDA receptor trafficking and properties [103]. Altogether, this suggests a complex regulation of VitB12 metabolism involving the NMDA receptors (Figure 4C).

Despite its pivotal role in sustaining cellular functions, VitB12 also acts as a metabolic cofactor for gut microbes. Due to its influence on microbial communities, VitB12 additionally impacts local and peripheral immunity, contributing to the concept known as gut-brain homeostasis. It is important to note that VitB12 not only takes part in the metabolism of host cells but also actively fosters the preservation of healthy gut microbiota and its associated metabolites, which, in turn, have an effect on the host. Therefore, these elements play a crucial role in maintaining a protective immune equilibrium, both under normal and disease conditions. Additionally, since there exists some evidence supporting the notion that VitB12 may alleviate inflammation associated with brain pathologies, this could introduce a new layer of intricacy to our understanding of the role of VitB12 in neuropathology [104].

### 4.2. Hyperhomocysteinemia and Neuropathology

It is worth emphasizing that the brain is particularly susceptible to oxidative stress due to its high oxygen consumption, low antioxidant capacity, and a significant concentration of polyunsaturated fatty acids that are prone to lipid peroxidation [105]. Although VitB12 deficiency is known to be associated with a high oxidative stress status, there is a question about whether the accumulation of HCy due to a deficiency in VitB12 might contribute to oxidative stress buildup in the brain. Indeed, it is reported that even healthy individuals can show low levels of folate and VitB12, and also considerable amounts of HCy, leading to oxidative stress accumulation and inflammation [106]. In mice, the high level of HCy (hyperhomocysteinemia) determines cognitive impairment linked to synaptic remodeling of cortex neurons and induces a decreased expression of the transcription factors HES1 and HES5, which play a role in neuronal regeneration [107]. Interestingly, Zhang and co-workers reported that high concentrations of HCy reduce mitochondrial spare respiration capacity without affecting mitochondrial membrane potential and, surprisingly, lower the production of ROS in neural cell lines (cortical neuron cell line RN-c and mouse hippocampal neuron cell line HT22) [108]. In accord with this, in adult rats, hyperhomocysteinemia leads to the manifestation of anxiety-like symptoms, memory problems, and hippocampal atrophy without affecting ROS production [109]. Moreover, an excessive accumulation of HCy has been shown to trigger ROS-independent apoptosis in NSC-34D cells (a hybrid cell line of motor neurons), achieved through the activation of caspases 3 and 7 [110]. Therefore, these findings suggest an important role of HCy in neuropathology, potentially acting as a toxic agent, although the precise and possible relationship between hyperhomocysteinemia and oxidative stress remains to be clarified.

### 4.3. A Possible Role of VitB12 in the Modulation of Gene Expression

Although many speculations exist regarding the possible biochemical modifications that can lead to molecular changes, detailed mechanisms regarding epigenetic modifications and the possible involvement of VitB12 remain yet to be fully elucidated. As previously mentioned, a deficiency in VitB12 can yield a direct impact, such as diminishing the SAM and succinate pool, but also an indirect one by exacerbating cellular oxidative stress. This oxidative stress has the dual effect of deactivating certain elements, like histone deacetylase 2 (HDAC2), while concurrently activating the DNA methylating enzyme, DNMT1 [111]. Experimental results have pointed out that supplementary treatment involving CN-Cbl leads to increased genome-wide DNA methylation and decreased expression of proinflammatory genes, such as IL-1β and CCL3, in neonatal rats affected by pneumococcal meningitis [112]. This suggests a potential role of VitB12 in regulating inflammation through an epigenetic mechanism.

Recently, we have examined various RNA-seq datasets derived from the cells of the brain or nervous system of mice or rats under VitB12 deficiency conditions. Surprisingly, despite the considerable heterogeneity in terms of biological material and experimental protocols employed to induce VitB12 deficiency, an alteration in the expression levels of genes encoding ribosomal proteins was observed similar in all the animal models considered, suggesting the existence of a basic conserved underlying mechanism [113].

Zhong and collogues evaluated the protective role of VitB12 against oxidative stress induced by H_2_O_2_ treatment by studying changes in protein expression levels in an SH-SY5Y [114]. While this study focuses on proteins, it is conceivable that the majority of alterations in the protein pool result from modifications in gene expression. On comparing the cells treated with H_2_O_2_ and the cells treated with H_2_O_2_ followed by recovery in VitB12, 22 proteins were observed to be differently modulated. In particular, 12 proteins (MT1F, SUMO3, AKAP8L, PSMG4, DHFR, PYCR1, ALKBH3, DUS3L, PTBP1, APAF1, HSP90AB4P, MRGBP) were upregulated and 10 proteins (PRKCSH, KHDRBS1, NDUFA5, ANLN, CPSF3L, NME2, COL5A2, TBCEL, DSP, DPM3) were downregulated. The fact that these proteins exhibited both upregulation and downregulation indicates the existence of a complex cellular response to the treatment with VitB12. To provide a better understanding of the molecular mechanisms associated with the protective effects of VitB12, the researchers performed a network analysis using Cytoscape 3.10.1 (an open-source software platform used for visualizing, analyzing, and modeling complex biological networks). They constructed an integrated regulatory network that included differentially expressed proteins and their interaction targets derived from various databases. This network consisted of 614 nodes and 712 edges, providing a comprehensive view of the protein–protein interactions associated with VitB12 regulation. Furthermore, a gene ontology analysis was performed, and it highlighted robust associations between the genes influenced by VitB12 and essential biological processes, including the cellular macromolecule metabolic process, cellular and nucleic acid metabolic processes, and gene expression. Among the differentially expressed proteins, PTBP1, a protein involved in pre-mRNA processing, stood out as it was upregulated in VitB12-treated cells. PTBP1 has a crucial role in neuroprotection because the PTBP1 knockdown completely abolishes VitB12 neuroprotection [114]. Furthermore, it is intriguing to observe that PTBP1 is closely associated with pre-mRNAs within the nucleus, and its involvement extends to the regulation of pre-mRNA processing, as well as other facets of mRNA metabolism and transport. This could suggest the potential role of VitB12 in modulating the transcriptome by possibly influencing the alternative splicing mechanisms.

### 4.4. The Protective Role of Vitamin B12: A Story Told by Neuronal Diseases

**Figure 6:**
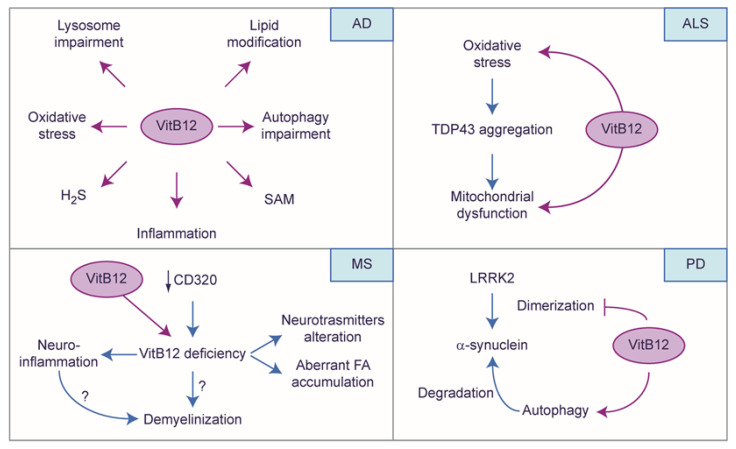
Many insights into the function of Vitamin B12 have emerged from conditions that commonly deteriorate due to its deficiency. The outline illustrates the more recent findings regarding Alzheimer’s Disease (AD), Amyotrophic Lateral Sclerosis (ALS), Multiple Sclerosis (MS), Parkinson’s Disease (PD), and Vitamin B12 deficiency. The question mark indicates that the mechanism has been poorly characterized. The dark blue arrows signify a decrease in CD320. Purple arrows represent involved processes, while the blunt-ended line denotes an inhibitory effect.

Numerous insights into the role of VitB12 have been derived from pathologies that usually worsen due to VitB12 deficiency (Figure 6). As mentioned previously, oxidative stress is a feature of neurodegenerative diseases, and VitB12 can induce antioxidant effects by participating indirectly in the biosynthesis of glutathione and H_2_S [115]. Interestingly, oxidative stress is a risk factor for Alzheimer’s disease (AD) [116], a disease condition marked by inflammation, oxidative stress, and reduced levels of SAM and H_2_S [117]; these symptoms could highlight a potential connection with VitB12 metabolism. Furthermore, various post-mortem analyses of the AD brain tissue have revealed significant lipid modifications, including changes in the total phospholipids, sphingomyelin, ceramide, and particularly, plasmalogens [118,119]. Plasmalogens, with a vinyl ether bond at the sn-1 position of the glycerol backbone, are particularly susceptible to oxidation. The association between oxidative stress, lipid modification, and disease progression is intriguing, as the generation of Aβ occurs within the confinement of biological membranes [120]. Considering the evidence that suggests a link between VitB12 deficiency and neurodegenerative disease, including AD [121,122,123,124,125], Theiss and co-workers investigated the potential impact of VitB12 on cell lipid composition using SH-SY5Y as a model. Specifically, when the cells were treated with hydrogen peroxide (H_2_O_2_) to induce oxidative stress, the administration of VitB12 demonstrated a beneficial effect on the plasmalogens. This effect was attributed to the indirect antioxidant activity of VitB12, which upregulated the expression of superoxide dismutase (SOD) and catalase (CAT) enzymes responsible for degrading ROS [126]. A similar effect has been observed in melanocytes, where VitB12 (specifically Me-Cbl) can induce the activation of Nuclear factor erythroid 2-related factor 2 (NRF2), subsequently promoting the transcription of target genes *SOD* and *CAT*, and reducing oxidative stress triggered by H_2_O_2_ [127]. This observation is crucial and warrants investigation in nerve system models because NRF2 plays a pivotal role as the regulator of antioxidant defense [128], and its dysregulation has been increasingly noted in various neurodegenerative conditions [129,130].

In SH-SY5Y cells, VitB12 enhanced the synthesis of plasmalogens by promoting the expression of alkylglycerone phosphate synthase (AGPS) and choline phosphotransferase1 (CHPT1) [126], suggesting an impact of VitB12 on cell lipid composition. Interestingly, comparing the works of Theiss and Zhong (both were carried out using SH-SY5Y cells), it is to be noted that Theiss and coworkers employed VitB12 at a dose of 10 nM, which was indeed contrasting compared to the 20 mM concentration of VitB12 utilized by Zhong and co-workers. Considering the optimal VitB12 blood concentration, which ranges from 250 to 850 pM [56,57], it becomes apparent that the former study employed a concentration approximately 11 times higher than the upper limit observed in healthy individuals, whereas, the latter study utilized a concentration approximately 24,000,000 times higher. Although Zhong reported the absence of VitB12 toxicity, the high dose could bring to light the specific modifications not observable at the lower dose.

In AD, lysosomal function deteriorates due to defective lysosomal acidification and disrupted lysosomal proteolysis due to mutations in the AD-related PSEN1 gene. Interestingly, in the SH-SY5Y cell model engineered for expressing APP (SH-SY5Y-AβPP), which was treated with a proteasome inhibitor to induce lysosomal Aβ accumulation, the levels of lysosomal cobalamin doubled. Furthermore, in AβPPxPS1 transgenic AD mice, the lysosomal cobalamin levels in the brain were significantly increased by 56% compared to wild-type control mice. These findings provide evidence of impaired lysosomal cobalamin transport in AD, associated with amyloid-β accumulation [65], thereby suggesting that a cellular specific-compartmentation deficiency of VitB12 can arise in AD and exacerbate the disease itself. Moreover, HCy further induces dysfunction within the lysosomes and impairs autophagy in both human and mouse primary astrocyte cultures [131]. In the context of a scopolamine-induced AD rat model, observations within the hippocampus reveal that pre-treatment with VitB12 can not only effectively counter the decrease of neurexin 1 and neuroligin, and the elevation of COX-2 and activation of caspase-3 (markers employed to assess AD’s severity and progression) but also alleviate the decline of postsynaptic density protein 95 (PSD-95), a scaffold protein that regulates synaptic maturation and plasticity. These observations hypothesize the protective nature of VitB12 against hippocampal inflammation and apoptosis within the AD model induced by scopolamine. Furthermore, they also suggest a potential role in safeguarding the synaptic integrity within the AD model’s hippocampus [132].

A recent study has shown that oxidative stress-induced aggregates of TDP-43, associated with amyotrophic lateral sclerosis (ALS), cause mitochondrial dysfunction [133]. The potential protective role of VitB12 and its underlying mechanism were investigated by researchers in SH-SY5Y cells, which were overexpressing TDP-43 [134], and it was observed that mitochondrial toxicity induced by TDP-43 in SH-SY5Y cells led to disruptions in the mitochondrial membrane potential, intracellular calcium balance, reduced oxygen consumption, and decreased ATP production. However, these detrimental effects were mitigated when the cells were treated with OH-Cbl. Interestingly, OH-Cbl did not directly reduce the aggregation of TDP-43 but it effectively alleviated mitochondrial dysfunction. It was revealed that the alteration in TDP-43 expression activated the mitochondrial unfolded protein response (mtUPR) control system, which typically promotes cell survival and organelle repair. However, the excessive accumulation of TDP-43 in the mitochondria seemed to induce irreversible damage to the organelle itself. Moreover, OH-Cbl was observed to effectively suppress the upregulation of mtUPR genes, thereby attenuating the neurotoxicity induced by TDP-43, improving the mitochondrial function and oxidative stress [133].

In Parkinson’s disease (PD), the propagation of α-synuclein is associated with the mutation of leucine-rich repeat kinase 2 (LRRK2) [135]. Interestingly, Ado-Cbl acts as a mixed-type-allosteric inhibitor of LRRK2 by directly interacting with LRRK2, leading to significant alterations in the protein conformation and ATP binding within the LRRK2 structure. Thereby, Ado-Cbl disrupts the LRRK2 dimerization [136], possibly inhibiting the propagation of α-synuclein. Furthermore, in accordance with the previous observation, Cui and co-workers observed in the MPTP-induced PD mouse model that the inhibition of LRRK2 by Ado-Cbl resulted in restoring autophagy and, in turn, removed protein aggregations which could possibly aid the treatment of PD; in particular, they employed a Tetrahedral framework nucleic acid-VitB12 complex (TVC), which was aimed at overcoming possible limitations due to the blood–brain barrier and TC availability, obtaining good results on disease symptoms [137].

In the CNS of both EAE mice and multiple sclerosis (MS) patients, there is a significant downregulation of CD320. Interestingly, the *Cd320*^−/−^ mouse model develops VitB12 deficiency in the nervous system and neuropathology associated with VitB12 deficiency [138]. Thus, the low expression of CD320 observed in EAE mice and MS patients could potentially cause impaired VitB12 uptake within the CNS, although, according to Scalabrino and co-workers, no change in VitB12 content was detected in the blood and cerebrospinal fluid (CSF) of MS patients [95]. Furthermore, studies have shown that mice deficient in VitB12 exhibit significantly reduced VitB12 levels in their spinal cords, increased histological damage, and severe EAE manifestations. These findings highlight a plausible connection between VitB12 deficiency and the progression of pathology in EAE and MS [62,139]. Jonnalagadda and colleagues investigated the alterations in gene expression patterns of a specific type of astrocytes known as immediate-early astrocytes (ieAstrocytes). These astrocytes, which constitute more than 95% of c-Fos-activated cells during EAE, are involved in the pathogenesis and progression of the disease, and exhibit a proportional rise in numbers with increasing severity of EAE [140]. The analysis revealed the differentially expressed genes to be associated with proinflammatory cascades, thereby suggesting an inflammatory response in VitB12-deficient astrocytes. Interestingly, VitB12-deficient astrocytes exhibited a reactive astrocyte phenotype, even in the absence of inflammatory stimuli. Furthermore, VitB12 deficiency resulted in the downregulation of genes related to neuroprotective interferon type I (IFN-I) signaling pathways in astrocytes, indicating a reduced sensitivity of astrocytes to IFN-I (which plays a crucial role in neuroprotection). Moreover, a significant decrease in the expression of IFN-β, an endogenous ligand for IFN-I receptors, was also revealed in VitB12-deficient EAE spinal cords, and this downregulation could be possibly due to the microglia and other potential immune cell types within the affected CNS. These mechanisms contribute to the aggravation of EAE symptoms in VitB12-restricted animals, shedding light on the involvement of this vitamin in neuroinflammatory conditions generally associated with MS [63]. Remarkably, in humans, clinical findings and MRI images often exhibit similar results for VitB12 deficiency and MS, including cerebral white matter demyelination, posing a significant challenge in accurately distinguishing between the two very different diagnoses [21]. Also, within MS patients, a reduction in VitB12 levels has been observed [139]; however, at the clinical level, it is known that VitB12 is crucial for the initial myelination and development of the CNS, as well as sustain its regular functioning. Therefore, VitB12 deficiency can result in demyelination of the cervical and thoracic dorsal and lateral columns of the spinal cord, sporadic demyelination in cranial and peripheral nerves, and white matter demyelination in the brain [8]. Despite these observations, the precise mechanism by which VitB12 deficiency triggers demyelination remains elusive. At the biochemical level, it is conceivable that the deficiency of SAM hampers the methylation of basic myelin protein and lipids, which can be detrimental to the myelin sheath. Furthermore, the build-up of methylmalonyl-CoA can result in a decline in normal myelin synthesis and the incorporation of anomalous fatty acids into neuronal lipids [21,141]. VitB12 deficiency can also influence myelin homeostasis by modifying the activity of oligodendrocytes and modulating the inflammation resulting in axon demyelination, thereby resulting in neuron injury and degeneration [139,142]. Also, VitB12 deficiency has been correlated with increased levels of TNF-α, along with decreased levels of epidermal growth factor (EGF) and IL-6 in humans [93,143]. This immunopathological scenario could be a contributing factor to demyelination. Besides demyelination, VitB12 deficiency can also lead to the accumulation of abnormal fatty acids in neuronal cells and modification in the levels of some neurotransmitters [20,144,145]. Altogether, these events result in neurodegeneration causing cerebral dysfunction, brain atrophy, and dementia [20,144,146], and emphasize that biochemical decompensation resulting from VitB12 insufficiency is not the sole contributor to demyelination; secondary effects, linked to cellular and molecular mechanisms due to VitB12 deficiency, can play a pivotal role in maintaining neuronal homeostasis.

## 5. Conclusions

In this review, we aimed to rationalize the currently available information concerning VitB12 deficiency and its impact on the nervous system, highlighting the heterogeneity of systems employed to gather these results and the experimental complexity involved. Overall, a clear link emerges regarding the role of VitB12 in the development, maintenance, and proper functioning of the nervous system.

Despite VitB12 studies commencing as early as the 1960s, the full extent of the biochemical alterations remains incompletely elucidated to this day. Even less understood are the specific molecular and cellular changes that can result from these metabolic alterations, although it is possible to speculate based on our current understanding of physiological states.

As discussed, various experimental limitations hinder the comprehensive characterization regarding VitB12, such as inducing deficiency or, conversely, supplementation. Similarly, the genetic engineering of enzymes involved in VitB12 metabolism, for the study of deficiency, poses challenges, given that these enzymes appear to be engaged in other molecular activities beyond their enzymatic function or their use of VitB12 as a cofactor.

Focusing on the nervous system, several studies highlight how metabolic alterations due to VitB12 deficiency can trigger molecular/cellular changes, such as the accumulation of oxidative stress, epigenetic modifications, modulation of gene expression or protein content, or alterations in lysosomal activity. Although these results primarily pertain to cellular dimensions, the deterioration of the nervous system, evident at the tissue and organ levels, includes phenomena like inflammation and demyelination.

Overall, while clinical data strongly link VitB12 deficiency to neurological issues, our current understanding of the underlying cellular and molecular mechanisms remains limited. The pathological data presented here have been integrated with our knowledge of physiological functionality, allowing us to hypothesize a part of the total picture framework. Nevertheless, acknowledging this association provides a powerful motivation to propel supporting research that can identify the precise mechanisms and find new therapeutic targets aimed at restoring the nervous system impairments due to severe VitB12 deficiency, rather than approaching these disorders with cures that can just decelerate the decline.

## Figures and Tables

**Table 1:** Experimental models.

Experimental Models	Induction of VitB12 Deficiency	Treatment	Reference
Mouse	Cbi supplementation through osmotic minipumps	21 female mice, 27 days treatment	[48]
		- Group 1: 102 nmol/24 h of Cbi	
		- Group 2: 42 nmol/24 h of VitB12 (resulting in 2,000,000 times more concentrated respect to humans)	
		- Group 3: control	
Mouse	EtPhCbl supplementation through osmotic minipumps	18 female mice, 27 days treatment	[49]
		- Group 1: 3.5 nmol/24 h of EtPhCbl	
		- 3.5 nmol/24 h of VitB12 (CN-Cbl)	
		- Group 3: control	
Mouse	Ronin^F80L/F80L^ and Hcfc1^A115V/Y^ mouse model using CRISPR/Cas9 genome editing	/	[50]
Mouse	1. Mmachc^flox/flox^ mouse model generated using CRISPR/Cas9 genome editing	/	[37]
	2. Mmachc-OE^+/tg^ transgenic mouse line that over-expresses functional mmachc		
Rat	Rats made VitB12 deficient by total gastrectomy	/	[51]
Rat	Rats administered with N2O (which induces the irreversible oxidation of Co+ to the Co+++ form, rendering VitB12 inactive).	/	[52]
Zebrafish	Embryos carrying the c.95_132delins28 p.Gly32Valfs*48 mutation in the mmachc (hg13) gene in a homozygous state	/	[53]
Zebrafish	1. mmachc morphant mutant created using morpholinos or simRNA to target the mRNA of embryos	/	[54]
	2. Germinal mutant created using zinc finger nucleases (ZFNs) approach		

## Data Availability

Data is contained within the article.
